# Identification and Partial Characterization of a Novel UDP-*N*-Acetylenolpyruvoylglucosamine Reductase/UDP-*N*-Acetylmuramate:l-Alanine Ligase Fusion Enzyme from *Verrucomicrobium spinosum* DSM 4136^T^

**DOI:** 10.3389/fmicb.2016.00362

**Published:** 2016-03-23

**Authors:** Kubra F. Naqvi, Delphine Patin, Matthew S. Wheatley, Michael A. Savka, Renwick C. J. Dobson, Han Ming Gan, Hélène Barreteau, Didier Blanot, Dominique Mengin-Lecreulx, André O. Hudson

**Affiliations:** ^1^Thomas H. Gosnell School of Life Sciences, Rochester Institute of TechnologyRochester, NY, USA; ^2^Institute for Integrative Biology of the Cell, CEA, CNRS, Univ Paris-Sud, Université Paris-SaclayOrsay, France; ^3^Biomolecular Interaction Centre, School of Biological Sciences, University of CanterburyChristchurch, New Zealand; ^4^Department of Biochemistry and Molecular Biology, Bio21 Molecular and Biotechnology Institute, The University of MelbourneParkville, VIC, Australia; ^5^Monash University Malaysia Genomics Facility, Monash University MalaysiaSelangor, Malaysia; ^6^School of Science, Monash University MalaysiaSelangor, Malaysia

**Keywords:** MurB, MurC, UDP-*N*-acetylenolpyruvoylglucosamine reductase, UDP-*N*-acetylmuramate:l-alanine ligase, fusion enzyme, bacterial cell wall, peptidoglycan, *Verrucomicrobium spinosum*

## Abstract

The enzymes involved in synthesizing the bacterial cell wall are attractive targets for the design of antibacterial compounds, since this pathway is essential for bacteria and is absent in animals, particularly humans. A survey of the genome of a bacterium that belongs to the phylum Verrucomicrobia, the closest free-living relative to bacteria from the Chlamydiales phylum, shows genetic evidence that *Verrucomicrobium spinosum* possesses a novel fusion open reading frame (ORF) annotated by the locus tag (VspiD_010100018130). The ORF, which is predicted to encode the enzymes UDP-*N*-acetylenolpyruvoylglucosamine reductase (MurB) and UDP-*N*-acetylmuramate:l-alanine ligase (MurC) that are involved in the cytoplasmic steps of peptidoglycan biosynthesis, was cloned. *In vivo* analyses using functional complementation showed that the fusion gene was able to complement *Escherichia coli murB* and *murC* temperature sensitive mutants. The purified recombinant fusion enzyme (MurB/C_*Vs*_) was shown to be endowed with UDP-*N*-acetylmuramate:l-alanine ligase activity. *In vitro* analyses demonstrated that the latter enzyme had a pH optimum of 9.0, a magnesium optimum of 10 mM and a temperature optimum of 44–46°C. Its apparent *K*_*m*_ values for ATP, UDP-MurNAc, and l-alanine were 470, 90, and 25 μM, respectively. However, all attempts to demonstrate an *in vitro* UDP-*N*-acetylenolpyruvoylglucosamine reductase (MurB) activity were unsuccessful. Lastly, Hidden Markov Model-based similarity search and phylogenetic analysis revealed that this fusion enzyme could only be identified in specific lineages within the Verrucomicrobia phylum.

## Introduction

Bacteria belonging to the Verrucomicrobia phylum are Gram-negative heterotrophic organisms that are generally found in soil and fresh water environments. The phylum is considered to have two sister phyla, Chlamydiae and Lentisphaerae (Cho et al., 2004). Members of the Verrucomicrobia are of interest due to their close evolutionary relationship to bacteria from the genus Chlamydia in addition to their unusual morphology of possessing wart-like and tube-like appendages that protrude from the cell membrane, commonly referred to as prosthecae (Wagner and Horn, 2006; McGroty et al., 2013). Most of the research that has been done with bacteria from this phylum has been done using *Verrucomicrobium spinosum* as the model organism. The bacterium was found to employ the type III secretion system and is pathogenic toward *Drosophila melanogaster* and *Caenorhabditis elegans* (Sait et al., 2011). In addition, research from our group recently demonstrated that the bacterium employs the L,L-diaminopimelate aminotransferase (DapL) pathway for the synthesis of *meso-*diaminopimelate involved both in the cross-linking of peptidoglycan (PG) and in lysine anabolism (Nachar et al., 2012; McGroty et al., 2013). Due to the morphological complexity and unusual cellular plan of *V. spinosum*, the synthesis of PG is of interest to our group given its close relationship to the pathogenic organisms from the genus Chlamydia. In addition, the recent discovery of PG in Chlamydia has made this project more intriguing, given the fact that even though β-lactam antibiotics are effective against Chlamydia, definitive evidence of PG in Chlamydial species has been lacking until this recent discovery (Pilhofer et al., 2013; Packiam et al., 2015).

Cell wall PG (also named murein) is ubiquitous in the bacterial domain. The PG of bacteria is composed of tandem repeats of the sugars *N*-acetylglucosamine (GlcNAc) and *N*-acetylmuramic acid (MurNAc) cross-linked by a short peptide stem containing usually L-lysine or *meso*-diaminopimelate at the third position (Park, 1987; Vollmer et al., 2008). Due to its rigid structure and tensile strength, the PG has several overarching roles such as protecting the osmotic integrity of the cell in addition to maintaining the shape of the bacteria.

**Figure 1:**
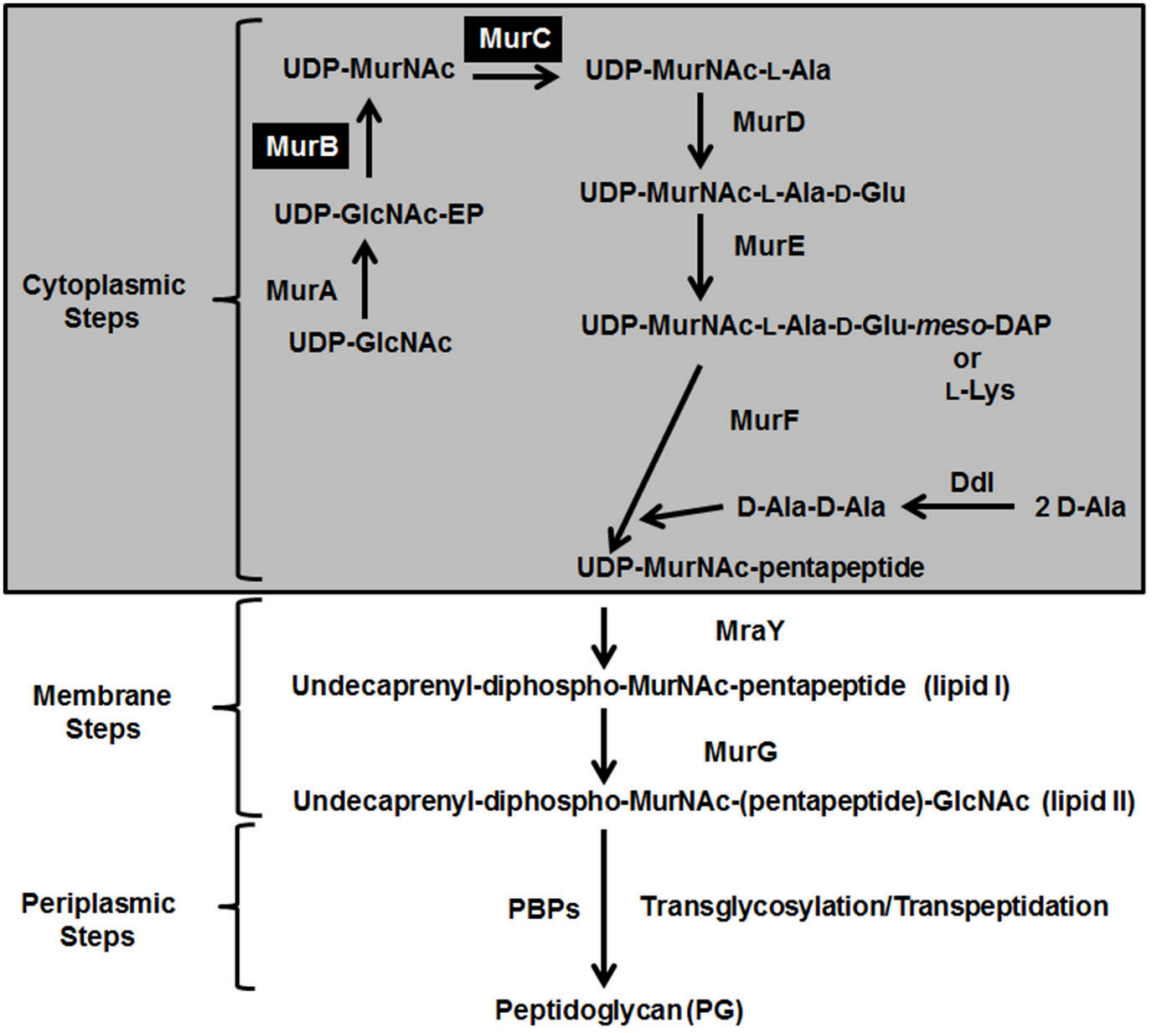
**Schematic representation depicting the three stages of PG biosynthesis in bacteria**. The cytoplasmic, membrane, and periplasmic sgteps are shown. The abbreviations of the enzymes are as follows: MurA, UDP-*N*-acetylglucosamine 1-carboxyvinyltransferase; MurB, UDP-*N*-acetylenolpyruvoylglucosamine reductase; MurC, UDP-*N*-acetylmuramate:l-alanine ligase; MurD, UDP-*N*-acetylmuramoyl-l-alanine:d-glutamate ligase; MurE, UDP-*N*-acetylmuramoyl-l-alanyl-d-glutamate:2,6-diaminopimelate ligase or UDP-*N*-acetylmuramoyl-l-alanyl-d-glutamate:l-lysine ligase; MurF, UDP-*N*-acetylmuramoyl-tripeptide:d-alanyl-d-alanine ligase; Ddl, d-alanine:d-alanine ligase; MraY, phospho-*N*-acetylmuramoyl-pentapeptide transferase; MurG, undecaprenyl-diphospho-*N*-acetylmuramoyl-pentapeptide β-*N*-acetylglucosaminyl transferase; and PBPs, penicillin-binding proteins. The enzymatic activities theoretically carried by the MurB/C fusion enzyme from *V. spinosum* are shaded in black. UDP-GlcNAc-EP stands for UDP-*N*-acetylenolpyruvoylglucosamine.

The synthesis of PG in bacteria occurs *via* a pathway that has three distinct steps: the cytoplasmic, membrane, and periplasmic steps. In the cytoplasmic steps, the nucleotide sugar-linked precursor UDP-MurNAc-pentapeptide is synthesized in a series of reactions catalyzed by the enzymes MurA, MurB, MurC, MurD, MurE, MurF, and Ddl (Figure 1) (Barreteau et al., 2008). The next steps in PG formation are the synthesis of the lipid precursor intermediates, undecaprenyl-diphospho-MurNAc-pentapeptide (lipid I) and undecaprenyl-diphospho-MurNAc-(pentapeptide)-GlcNAc (lipid II), by the enzymes MraY and MurG, respectively; these reactions occur at the level of the cytoplasmic membrane (Bouhss et al., 2008). The ultimate steps in the pathway are the transglycosylation and transpeptidation reactions, characterized by the polymerization of the sugar-peptide units and their incorporation into the growing PG; these reactions take place in the extra-cytoplasmic space and are catalyzed by the penicillin-binding proteins (PBPs) (Figure 1) (Scheffers and Pinho, 2005).

**Figure 2:**
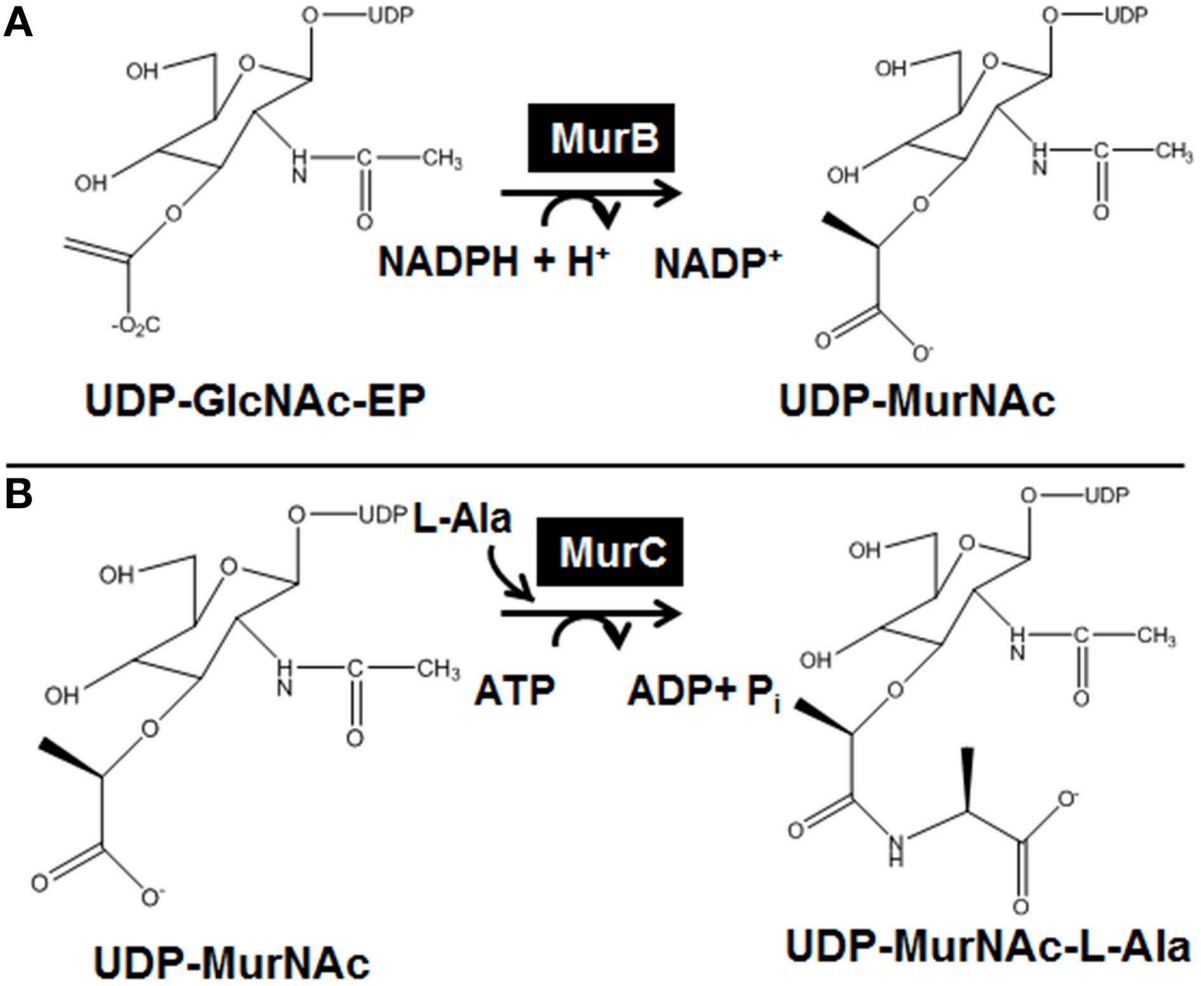
**Enzymatic reactions catalyzed by the MurB/C fusion enzyme**. **(A)** Reaction catalyzed by UDP-*N*-acetylenolpyruvoylglucosamine reductase (MurB). **(B)** Reaction catalyzed by UDP-*N*-acetylmuramate: l-alanine ligase (MurC).

The first three cytoplasmic steps of the PG synthesis pathway, which are the topic of this paper, are as follows. First, UDP-*N*-acetylglucosamine-1-carboxyvinyltransferase (MurA, EC 2.5.1.7) catalyzes the transfer of the enolpyruvyl moiety from phosphoenolpyruvate to the 3′-hydroxyl end of UDP-GlcNAc to produce UDP-*N*-acetylenolpyruvoylglucosamine (UDP-GlcNAc-EP) (Figure 1) (Marquardt et al., 1992; Wanke et al., 1992). Then, UDP-*N*-acetylenolpyruvoylglucosamine reductase (MurB, EC 1.3.1.98) catalyzes the reduction of the enolpyruvyl moiety of UDP-GlcNAc-EP to lactyl ether to produce UDP-*N*-acetylmuramic acid (UDP-MurNAc) (Figure 2A) (Benson et al., 1993; Tayeh et al., 1995). Finally, UDP-*N*-acetylmuramate:l-alanine ligase (MurC, EC 6.3.2.8) catalyzes the third reaction, which consists in the addition of L-Ala to the carboxyl group of UDP-MurNAc to produce UDP-MurNAc-l-Ala (Figure 2B) (Liger et al., 1995; Falk et al., 1996; Gubler et al., 1996).

Here we report the identification and biochemical partial characterization of a novel MurB/C fusion enzyme from *V. spinosum*. While *in vitro* assays demonstrate that the enzyme is able to catalyze the ligase (MurC) reaction, attempts to demonstrate the reductase (MurB) activity *in vitro* were unsuccessful. Nevertheless, *in vivo* analyses demonstrated that the fusion gene is able to functionally complement two *Escherichia coli* strains that harbor mutations in the *murB* and *murC* genes. Given the facts that (i) the MurB and MurC enzymes are not normally fused and are encoded by separate ORFs as is the case of the two *E. coli* proteins (Pucci et al., 1992; Liger et al., 1995), and (ii) the PG biosynthesis pathway is essential and is only present in the bacterial domain, the identification and characterization of this unusual fusion enzyme involved in PG biosynthesis in *V. spinosum*, a close relative of the pathogenic organism Chlamydia, is intriguing. This study has the potential to contribute to the further understanding of the kinetic, physical and structural properties of enzymes involved in the synthesis of PG in order to facilitate the development and/or discovery of antibacterial compounds that are able to combat current and emerging bacterial infections and diseases, especially those that are deemed to be resistant to antibiotics that are currently used in a clinical setting.

## Materials and methods

### Materials

l-[^14^C]Ala (5.99 GBq.mmol^−1^) and l-[^14^C]Ser (6.07 GBq.mmol^−1^) were purchased from Perkin Elmer, [^14^C]Gly (3.88 GBq.mmol^−1^) from CEA, and UDP-[^14^C]GlcNAc (2 GBq.mmol^−1^) from ARC Isobio. UDP-[^14^C]MurNAc was prepared according to published procedure (Bouhss et al., 2002). UDP-GlcNAc-EP was purchased from the BaCWAN facility.

### *V. spinosum* growth conditions/plasmids and strains used in this study

*V. spinosum* DSM 4136^T^ was cultured in R2A medium supplemented with 5% (w/v) artificial sea water at 26°C (Schlesner, 1987). The plasmids and strains used in this study are listed in Table 1.

**Table 1:** **Plasmids and strains used in this study**.

**Plasmids and strains**	**Sources/References**
**PLASMIDS**	
pET100D/topo	Invitrogen, USA
pBAD33	Guzman et al., 1995
pET100D::*murB/C_*Vs*_*	This study
pBAD33::*murB/C_*Vs*_*	This study
p*Trc*His60	Pompeo et al., 1998
p*Trc*His60::*murB_*Vs*_*-1	This study
p*Trc*His60::*murB_*Vs*_*-2	This study
p*Trc*His60::*murC_*Vs*_*-1	This study
p*Trc*His60::*murC_*Vs*_*-2	This study
p*Trc*His60::*murC_*Ec*_*	Liger et al., 1995
**STRAINS**	
*Verrucomicrobium spinosum* DSM 4136^*T*^	Schlesner, 1987
5-alpha competent cells	New England Biolabs, USA
Rosetta codon plus RIPL	Agilent, Technologies, USA
Rosetta (DE3) pLysS	Novagen, USA
*E. coli murB* (ST5) CGSC Strain # 6442	Matsuzawa et al., 1969; Miyakawa et al., 1972
*E. coli murC* (ST222) CGSC Strain # 5988	Miyakawa et al., 1972
*E. coli murC* (H1119)	Wijsman, 1972
*E. coli murB* (pBAD33)	This study
*E. coli murC* (pBAD33)	This study
*E. coli murB* (pBAD33::*murB/C_*Vs*_*)	This study
*E. coli murC* (pBAD33::*murB/C_*Vs*_*)	This study

### Domain mapping of MurB/C*_*Vs*_* identification

The protein family (Pfam) domains of MurB/C_*Vs*_ were identified using the Pfam server (http://pfam.sanger.ac.uk/) and (http://pfam.janelia.org/) (Finn et al., 2014). The conserved domain database (CDD) (http://www.ncbi.nlm.nih.gov/Structure/cdd/cdd.shtml) was used to identify the domains of MurB/C_*Vs*_ (Marchler-Bauer et al., 2015).

### Identification of lineages containing fused MurB/C protein and phylogenetic analysis of MurB and MurC proteins

Publicly available draft and complete genome sequences belonging to members of the phylum Planctomycetes, Verrucomicrobia, Chlamydiae, and Lentisphaerae (PVC) were downloaded from NCBI. The proteomes were re-predicted with Prodigal version 2.6 (Hyatt et al., 2010) and then used for phylogenomic tree construction with PhyloPhlAN (Segata et al., 2013). The manually curated database of protein families known as TIGFAMs was used to identify MurB (TIGR00179) and MurC (TIGR00182) proteins (Haft et al., 2003). The predicted whole proteomes were subjected to similarity search based on the hidden Markov model (HMM) profiles TIGR00179 and TIGR00182 using HMMsearch (–cut_tc option) (Eddy, 2011), and species containing unusual composition of MurB and MurC, i.e., fused MurB/C, missing MurB, and/or missing MurC, were annotated in the constructed species tree. Additionally, the gene organization of contigs containing the fused *murB/C* open reading frame (ORF) was further analyzed with EasyFig version 2.1 (Sullivan et al., 2011). Additional MurB and MurC proteins were also mined from the UniProt (http://www.uniprot.org/) (The UniProt Consortium) to be included for phylogenetic analysis. Briefly, the proteins were aligned and trimmed using mafft-linsi and trimal (-gappyout setting) (Capella-Gutiérrez et al., 2009; Katoh and Standley, 2013), and phylogenetic inference was performed using IQ-TREE version 1.3.10 (Nguyen et al., 2014) and visualized using FigTree version 1.42 (http://tree.bio.ed.ac.uk/software/figtree/).

### PCR amplification and cloning of the *V. spinosum murB/C* open reading frame (ORF)

The ORF annotated by the locus tag (VspiD_010100018130) UDP-*N*-acetylenolpyruvoylglucosamine reductase /UDP-*N*-acetylmuramate:l-alanine ligase was amplified by PCR using the primers *murB/C*_*Vs*_-forward (5′-C ACC**ATG**AATCACGCCGTCGTCAGTTTGTTGAA G-3′) and *murB/C*_*Vs*_-reverse (5′-GTCGAC**CTA**TAGCGGAAG CGGTTCCTCTTCGCCAAT-3′). The underlined sequence represents the restriction enzyme site used to facilitate sub-cloning of the ORF while the bold sequences represent initiation and termination codons. The PCR reaction contained 12 pmol of forward and reverse primers, 1 mM MgSO_4_, 0.5 mM of each of the four deoxynucleotide triphosphates, 0.5 ng of genomic DNA and 1 unit of Platinum *Pfx* DNA polymerase (Invitrogen Corporation, Carlsbad, CA, USA). PCR conditions were: 1 cycle at 94°C for 2 min, followed by 30 cycles of 94°C for 15 s, 60°C for 30 s, and 72°C for 3 min. The *murB/C* PCR fragment was ligated into the plasmid pET100D/topo (Invitrogen Corporation, Carlsbad, CA, USA) to produce the plasmid pET100D::*murB/C*_*Vs*_. The recombinant protein encoded by this plasmid carries an N-terminal MRGS**HHHHHH**GMASMTGGQQMGRDLYDDDDKDHPFT additional sequence containing a hexa-histidine tag (bold) derived from the pET100D plasmid. To confirm the fidelity of the PCR reaction, the ORF was sequenced from pET100D using the T7 promoter primer, 5′-TAATACGACTCACTATAGGG-3′ and the T7 reverse primer, 5′-TAGTTATTGCTCAGCGGTGG-3′. The cloned *murB/C* ORF was 100% identical to the sequence deposited in the Integrated Microbial Genomes public database (http://img.jgi.doe.gov/cgi-bin/w/main.cgi).

### Cloning of *murC* and *murB* domains

The *murC* and *murB* domains of the MurB/C_*Vs*_ fusion protein were also cloned separately in expression vectors, as follows. The *murC* domain sequence (two different end points were chosen) was amplified by PCR by using *murC*_*Vs*_-forward (5′-GCGCTC**ATG**AATCACGCCGT CGTCAGTTTGTTGAAG-3′) as the forward primer and either *murC*_*Vs*_-reverse-1 (5′-GCGCAGATCTGCCTTCGCG ATTGAGCACCGTAGTGAG-3′) or *murC*_*Vs*_-reverse-2 (5′-GCGCAGATCTGACCGTGCC ACCGCCTTCGCGATTGAG-3′) as the reverse primer, designed to end the MurC domain at the Gly477 and Val481 residues, respectively. The underlined sequences correspond to introduced *Bsp*HI and *Bgl*II restriction sites and the *murC* gene initiation codon is indicated in bold. The two PCR products were digested by *Bsp*HI and *Bgl*II and inserted between the compatible *Nco*I and *Bgl*II sites of the expression vector p*Trc*His60 that allows expression of proteins with a C-terminal hexa-histidine tag under the control of the IPTG-inducible *trc* promoter (Pompeo et al., 1998). The two plasmids thus generated, p*Trc*His60::*murC*_*Vs*_-1 and p*Trc*His60::*murC*_*Vs*_-2, directed expression of Met1-Gly477 and Met1-Val481 fragments from the MurB/C_*Vs*_ fusion protein (770 residues in total), respectively, fused to a C-terminal tag extension consisting in Arg-Ser-His_6_.

Similarly, the *murB* domain sequence (two different starting points were chosen) was amplified by using either *murB*_*Vs*_-forward-1 (5′-G AAGCC**ATG**GGCACGGTCAAGCTCTATGAGCCGAT G-3′) or *murB*_*Vs*_-forward-2 (5′-GCGCTC**ATG**AAGCTCTATG AGCCGATGGCCAACCAC-3′) as the forward primer and *murB*_*Vs*_-reverse (5′-GCGCAGATCTTAGCGGAAG CGGTTCCTCTTCGCCAATG-3′) as the reverse primer. *Nco*I, *Bsp*HI and *Bgl*II restriction sites introduced in these sequences are underlined and the *murB* gene initiation codons are indicated in bold. The two PCR products were digested by *Nco*I or *Bsp*HI and *Bgl*II and inserted between the compatible *Nco*I and *Bgl*II sites of the vector p*Trc*His60. The two plasmids thus generated, p*Trc*His60::*murB*_*Vs*_-1 and p*Trc*His60::*murB*_*Vs*_-2, directed expression of the Gly479-Leu770 and Lys482-Leu770 fragments (preceded by a Met residue) from the MurB/C_*Vs*_ fusion protein, respectively, here too with a C-terminal tag consisting in Arg-Ser-His_6_.

### Expression and purification of recombinant MurB/C_*Vs*_

The *E. coli* Rosetta (DE3) pLysS strain (Novagen) was transformed with the plasmid pET100D::*murB/C*_*Vs*_ and grown at 37°C in 2YT medium containing 50 μg.mL^−1^ ampicillin and 25 μg.mL^−1^ chloramphenicol. An overnight pre-culture of the resulting strain was used to inoculate 2YT medium (2-liter cultures). The culture was incubated with shaking at 37°C. When the optical density reached 0.9, the temperature of the culture was decreased to 20°C and IPTG was added at a 1 mM final concentration. Growth was continued for 18 h at 20°C. Cells were harvested at 4°C and the pellet was washed with cold 20 mM phosphate buffer, pH 7.2, containing 1 mM dithiothreitol (buffer A). Bacteria were resuspended in buffer A (12 mL) and disrupted by sonication in the cold using a Bioblock Vibracell 72412 sonicator. The resulting suspension was centrifuged at 4°C for 30 min at 200,000 × *g* with a Beckman TL100 apparatus, and the pellet was discarded. The supernatant was kept at −20°C.

The His_6_-tagged protein was purified on Ni^2+^-nitrilotriacetate (Ni^2+^-NTA)-agarose following the manufacturer's recommendations (Qiagen). All procedures were performed at 4°C. The supernatant was mixed for 1 h with the polymer previously washed with buffer A containing 0.3 M KCl and 10 mM imidazole. Washing and elution steps were performed with a discontinuous gradient of imidazole (20–300 mM) in buffer A containing 0.3 M KCl. Protein contents were analyzed by sodium dodecyl sulfate-polyacrylamide gel electrophoresis (SDS-PAGE) and relevant fractions were pooled and dialyzed against 100 volumes of buffer A. At this step, precipitation of a significant part of the protein was observed. The non-precipitated protein was concentrated on an Amicon Ultra 50,000 molecular mass cutoff filter. Glycerol (10% final concentration) was added for storage at −20°C. Protein concentrations were determined by quantitative amino acid analysis with a Hitachi L8800 analyzer (ScienceTec) after hydrolysis of samples at 105°C for 24 h in 6 M HCl containing 0.05% 2-mercaptoethanol. No attempts were made to remove the additional sequence containing the hexa-histidine tag after protein purification.

### Construction of the plasmid to facilitate functional complementation

The plasmid used for functional complementation of the *E. coli murB* and *murC* mutants was produced by sub-cloning the *Xba*I and *Sal*I fragment from the pET100D::*murB/C*_*Vs*_ plasmid into pBAD33 to produce the plasmid pBAD33::*murB/C*_*Vs*_, which is under control of the arabinose inducible promoter (Guzman et al., 1995). The protein produced from the pBAD33 construct is identical to the protein produced from the pET100D construct. Note that there is an *XbaI* site 20 base pairs upstream of the ribosome binding site of the pET100D vector that was used to facilitate sub-cloning from pET100D into pBAD33.

### Functional complementation of the *E. coli murB* and *murC* thermosensitive mutants

The *E. coli murB* mutant (ST5-strain #6442) [thr-1, araC14, leuB6 (Am), secA216, fhuA61, lacY1, galT23, λ^−^ trp-84, his-215, thyA710, rpsL263 (strR), xylA5, mtl-1, murB1-(ts), thi-1] and murC mutant (ST222-strain #5988) [thr-1, araC14, leuB6 (Am), murC3(ts), secA216, fhuA61, lacY1, galT23, λ^−^ trp-84, his-215, thyA710, rpsL263 (strR), xylA5, mtl-1, thi-1] were both obtained from the Coli Genetic Stock Center (http://cgsc.biology.yale.edu/). These mutants were transformed with the pBAD33 vector or the pBAD33::*murB/C*_*Vs*_ plasmid and transformants were selected on LB agar medium supplemented with 50 μg.mL^−1^ thymine, 34 μg.mL^−1^ chloramphenicol, and 0.2% (w/v) arabinose at 30°C. Colonies were then replica-plated onto LB agar medium plus 0.2% (w/v) arabinose and 10 μg.mL^−1^ thymine. Liquid cultures were also performed in LB medium supplemented with arabinose and thymine. In both cases, the growth phenotype was assessed at 30 and 42°C for up to 24 h.

As described above, the individual *murC* and *murB* domains from the *murB*/*C*_*Vs*_ fusion gene were also cloned separately in the p*Trc*His60 vector that allows high-level gene expression under control of the strong IPTG-dependent *trc* promoter. The resulting plasmids p*Trc*His60::*murB*_*Vs*_ and p*Trc*His60::*murC*_*Vs*_ were then tested for functional complementation, using the *E. coli murB* mutant ST5 and the *murC* mutant strain H1119 (Wijsman, 1972), respectively. Transformants were selected on LB agar medium supplemented with 100 μg.mL^−1^ ampicillin and 50 μg.mL^−1^ thymine at 30°C and were subsequently replicated on similar plates incubated at 30°C or 42°C, in the presence or absence of 0.5 mM IPTG. Growth was observed after 24 h of incubation.

### Determination of the kinetic constants of the MurC activity

The standard MurC activity assay (Liger et al., 1995) measured the formation of UDP-MurNAc-l-Ala in a mixture (final volume, 40 μL) containing 100 mM Tris-HCl, pH 9.0, 10 mM MgCl_2,_ 3 mM ATP, 10 mM ammonium sulfate, 0.5 mg.mL^−1^ bovine serum albumin (BSA), 0.9 mM UDP-MurNAc, 0.3 mM l-[^14^C]Ala (400 Bq), and enzyme (20 μL of an appropriate dilution in buffer A). For the determination of the *K*_*m*_ values for UDP-MurNAc, UDP-[^14^C]MurNAc was used as the radiolabelled substrate.

In all cases, the mixtures were incubated for 30 min at 37°C and the reactions were stopped by the addition of glacial acetic acid (10 μL) followed by lyophilization. Radioactive substrate and product were then separated by HPLC on a Nucleosil 100C_18_ 5 μm column (150 × 4.6 mm; Alltech France) using 50 mM ammonium formate, pH 3.2, at a flow rate of 0.6 mL.min^−1^. Radioactivity was detected with a flow detector (model LB506-C1, Berthold) using the Quicksafe Flow 2 scintillator (Zinsser Analytic) at 0.6 mL.min^−1^. Quantification was performed with the Radiostar software (Berthold).

For the determination of the kinetic constants, the same assay was used with various concentrations of one substrate and fixed concentrations of the others. In all cases, the enzyme concentration was chosen so that substrate consumption was < 20%, the linearity being ensured within this interval even at the lowest substrate concentration. Data were fitted to the equation *v* = *V*_*max*_*S*/(*K*_*m*_ + *S*) by the Levenberg-Marquardt method (Press et al., 1986), where *v* is the initial velocity and *S* is the substrate concentration, and values ± standard deviation at 95% of confidence were calculated. The MDFitt software developed by M. Desmadril (I2BC, Orsay, France) was used for this purpose.

### *In vitro* spectrophotometric assay of the MurB activity

The MurB spectrophotometic assay was performed as described previously (Benson et al., 1993). The reaction mixture contained, in a final volume of 100 μL, 50 mM Tris-HCl, pH 8.0, 20 mM KCl, 0.5 mM dithiothreitol, 0.1 mM UDP-GlcNAc-EP, and 0.15 mM NADPH. The mixture was placed in a 1- cm path length cell and the reaction was started by the addition of the enzyme. The decrease in NADPH absorbance at 340 nm was monitored with a Jasco V-630 spectrophotometer.

### *In vitro* coupled assay of the MurA/MurB activities

The reaction mixture contained, in a final volume of 40 μL, 50 mM Tris-HCl, pH 7.6, 25 mM KCl, 0.1 mM NADPH, 55 μM UDP-[^14^C]GlcNAc (500 Bq), 75 μM phosphoenolpyruvate, *E. coli* MurA (1 μg), and enzyme. In some experiments, 5 mM ATP, 10 mM MgCl_2_, and 0.15 mM l-Ala were included. After 30 min at 37°C, the reaction was stopped by the addition of glacial acetic acid (8 μL) followed by lyophilization. The radioactive substrate and product were separated on a Nucleosil 100C_18_ 5 μm column (150 × 4.6 mm; Alltech France) using 50 mM ammonium formate, pH 3.2, at a flow rate of 0.6 mL.min^−1^. Detection and quantification of the radioactivity were performed as described above. The retention times for UDP-GlcNAc, UDP-GlcNAc-EP, UDP-MurNAc, and UDP-MurNAc-l-Ala were 6, 10, 12, and 20 min, respectively.

### MurB/C*_*Vs*_* cleavage assay

For the cleavage assay, *V. spinosum* was grown in liquid medium R2A medium supplemented with 5% (w/v) artificial sea water at 26°C for 5 days. Following centrifugation, the cells were lysed by sonication in the following buffer systems 50 mM Tris-HCl, pH 7.6, 50 mM Tris-HCl, pH 8.5, and 50 mM HEPES-KOH, pH 7.6. The purified recombinant enzyme (7.5 μg) was incubated with 15 μg of *V. spinosum* extract at 30°C. The proteins were resolved on a 10% (w/v) acrylamide gel and stained with Coomassie blue for visualization. Protein concentration was measured using the Bradford assay with BSA as the standard (Bradford, 1976).

## Results

### Identification of the MurB/C fusion enzyme from *V. spinosum*

The complete set of genes in *V. spinosum* required for the *de novo* synthesis of PG was initially identified from a comparative analysis of the *V. spinosum* proteome using the known PG biosynthesis proteins as queries (Nachar et al., 2012). The search revealed an anomaly when it was realized that both the MurB and MurC proteins were encoded by a single locus tag VspiD_010100018130 (Table 2).

**Table 2:** **List of PG biosynthesis genes from ***V. spinosum*****.

**Protein symbol**	**Gene product name**	**EC #**	**Locus Tag**
MurA	UDP-*N*-acetylglucosamine 1-carboxyvinyltransferase	2.5.1.7	VspiD_010100011745
**MurB**	**UDP-*****N*****-acetylenolpyruvoylglucosamine reductase**	**1.3.1.98**	**VspiD_010100018130**
**MurC**	**UDP-*****N*****-acetylmuramate:l-alanine ligase**	**6.3.2.8**	**VspiD_010100018130**
MurI	Glutamate racemase	5.1.1.3	VspiD_010100008415
MurD	UDP-*N*-acetylmuramoyl-l-alanine:d-glutamate ligase	6.3.2.9	VspiD_010100019115
MurE	UDP-*N*-acetylmuramoyl-l-alanyl-d-glutamate:2,6-Diaminopimelate ligase	6.3.2.13	VspiD_010100019130
MurF	UDP-*N*-acetylmuramoyl-tripeptide:d-alanyl-d-alanine ligase	6.3.2.10	VspiD_010100019125
AlaR	Alanine racemase	5.1.1.1	VspiD_010100000100
Ddl	d-alanine:d-alanine ligase	6.3.2.4	VspiD_010100018175
MraY	Phospho-*N*-acetylmuramoyl-pentapeptide transferase	2.7.8.13	VspiD_010100019120
MurG	Undecaprenyl-diphospho-*N*-acetylmuramoyl-pentapeptide β-*N*-acetylglucosaminyl transferase	2.4.1.227	VspiD_010100019100
UppP	Undecaprenyl-diphosphate phosphatase	3.6.1.27	VspiD_010100026230
PBP	d-alanyl-d-alanine carboxypeptidase-class C	3.4.16.4	VspiD_010100024635
PBP	Multimodular transpeptidase-transglycosylase-class A	2.4.1.129	VspiD_010100022270
PBP	Penicillin-binding protein 1C- class A	2.4.1.129	VspiD_010100006740
PBP	Penicillin-binding protein 2-class A	2.4.1.129	VspiD_010100020475
PBP	Peptidoglycan transpeptidase-class B	2.4.1.129	VspiD_010100007940
PBP	Peptidoglycan transpeptidase-class B	2.4.1.129	VspiD_010100017450
PBP	Peptidoglycan synthetase FtsI-class B	2.4.1.129	VspiD_010100019135
PBP	Cell elongation specific d,d-transpeptidase- class B	2.4.1.129	VspiD_010100018680

*The information for MurB and MurC is in bold. EC # denotes enzyme classification number*.

### Domain mapping of the MurB/C fusion enzyme from *V. spinosum*

**Figure 3:**
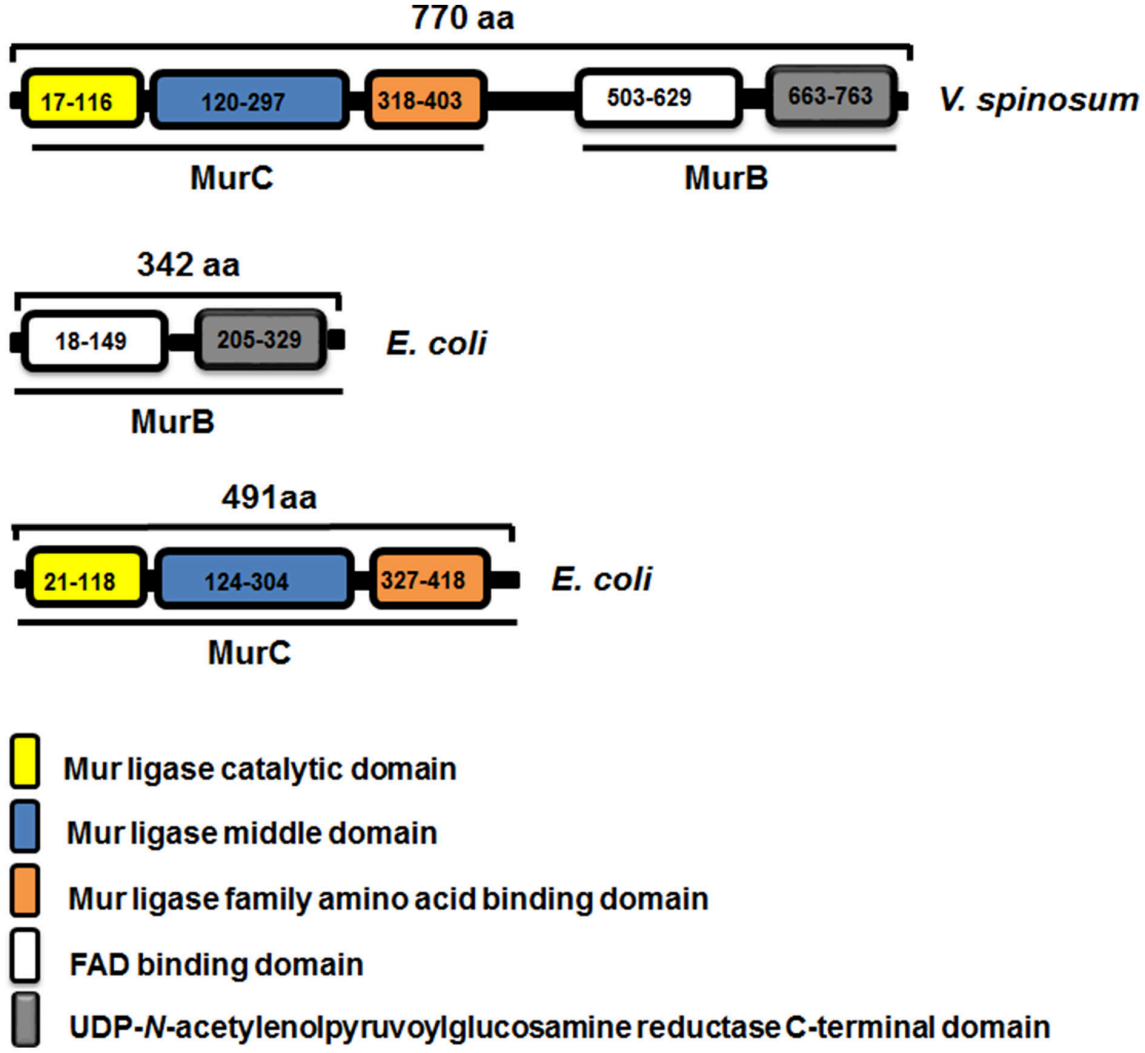
**Domain mapping of the MurB/C_***Vs***_ fusion enzyme of ***V. spinosum*** and the individual MurB and MurC enzymes from ***E. coli*** showing the predicted Pfam domains and residue locations of the individual domains**.

The length of the MurB and MurC *E. coli* proteins are 342 and 491 residues, respectively, and the lengths of the MurB/C fusion enzyme protein is 770 residues. As such, we were interested to assess if the fusion enzyme had the domains that are indicative of typical MurB and MurC enzymes. Domains were identified using the NCBI's CDD and the protein families database (Pfam) (Finn et al., 2014; Marchler-Bauer et al., 2015). The CDD and Pfam analyses resulted in the identification of the following domains: (1) the Mur ligase catalytic domain (Pfam01225), (2) the Mur ligase middle domain (Pfam08245), (3) the Mur ligase family amino acid-binding domain (Pfam02875), (4) the FAD-binding domain (Pfam01565), and (5) the UDP-*N*-acetylenolpyruvoylglucosamine reductase C-terminal domain (Pfam02873). This analysis also demonstrated that the residues responsible for the MurC activity were located toward the N-terminal end of the fusion enzyme, while those for the MurB activity are located toward the C-terminal end. MurC and MurB are separated by a linker region of ~100 residues as depicted in the schematic. For comparison, the figure also depicts the domain structures of the *E. coli* MurB and MurC enzymes (Figure 3).

### The *murB/C_*Vs*_* gene is able to functionally complement the *E. coli murB* and *murC* mutants

**Figure 4:**
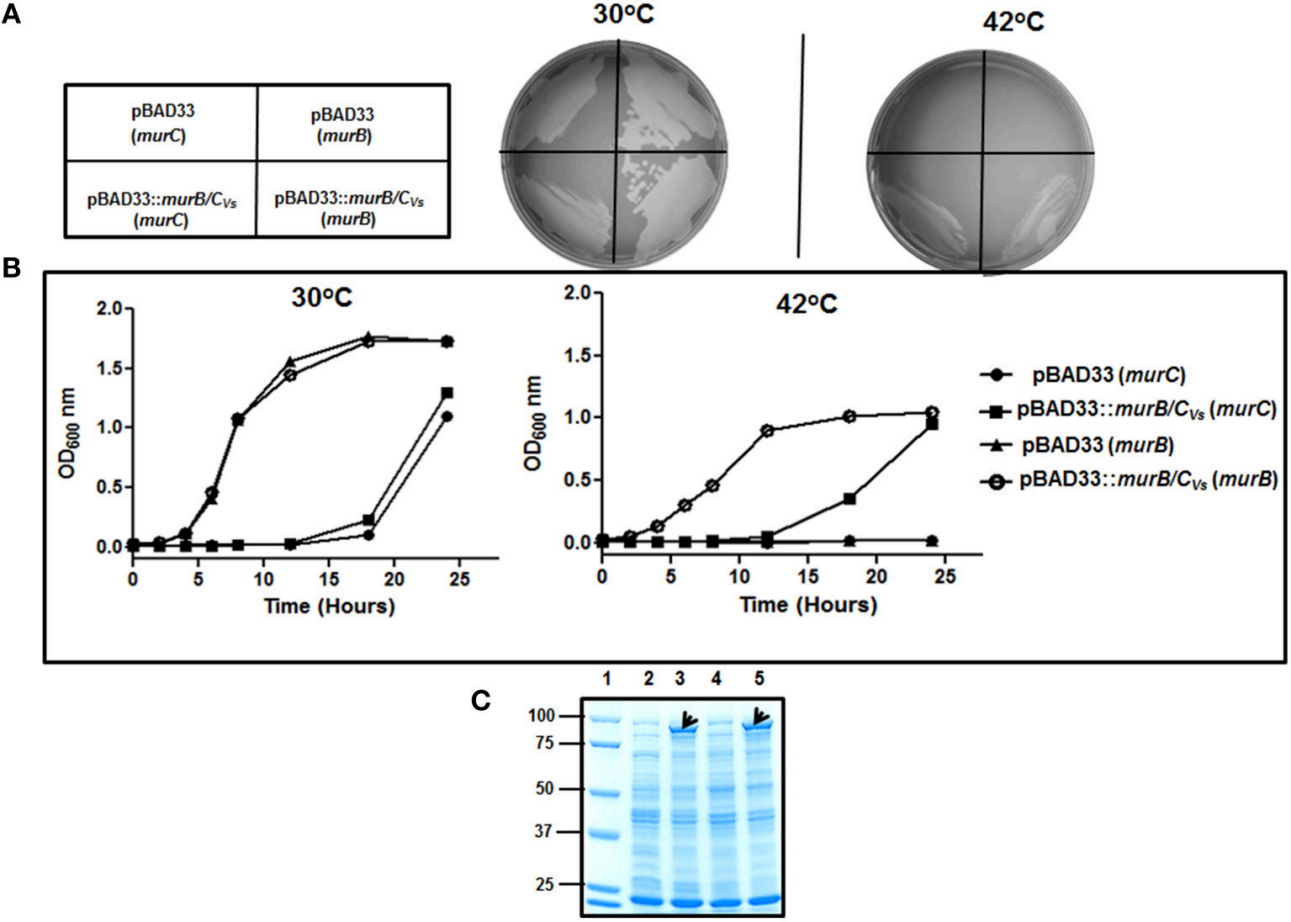
**Functional complementation analysis of the ***E. coli murB*** and ***murC*** mutants. (A)** Replica-plating experiment of the *murC* and *murB* mutants transformed with pBAD33 and pBAD33::*murB/C*_*Vs*_ grown at 30 and 42°C. **(B)** Analysis of the complementation experiment at 30 and 42°C in liquid culture assessing the growth phenotype by measurement of the optical density (OD) at 600 nm for a 24 h period. The growth experiments were done four times giving the similar growth profiles. The graphs in **(B)** represent one of those growth curves. **(C)** SDS-PAGE analysis of proteins from the complementation experiment to assess the expression of MurB/C in the mutant backgrounds. Lane (1), protein ladder (kDa). Lane (2), 10 μg of protein extract from the *E. coli murC* mutant harboring pBAD33 grown at 30°C. Lane (3), 10 μg of protein extract from the *E. coli murC* mutant harboring pBAD33::*murB/C*_*Vs*_grown at 42°C. Lane (4), 10 μg of protein extract from the *E. coli murB* mutant harboring pBAD33 grown at 30°C. Lane (5), 10 μg of protein extract from the *E. coli murB* mutant harboring pBAD33::*murB/C*_*Vs*_grown at 42°C. Crude extracts were obtained via sonication after 24 h from the samples grown at 30°C and 42°C. The black arrows show expression of the MurB/C recombinant enzyme. The proteins were resolved on a 10% (w/v) acrylamide gel and stained with Coomassie blue for visualization.

The *E. coli* strains ST5 and ST222 obtained from the Coli Genetic Stock Center harbor mutations in the *murB and murC* genes, respectively. These mutations result in a temperature-sensitive growth phenotype where the mutants are able to grow at the permissive temperature of 30°C, but not at the non-permissive temperature of 42°C (Matsuzawa et al., 1969; Miyakawa et al., 1972). To answer the question of whether the fusion gene is able to complement the *murB* and *murC E. coli* mutants, the mutant strains were transformed with an empty vector (pBAD33) or with a vector containing the *murB/C* gene (pBAD33::*murB/C*_*Vs*_). Using replica-plating, the results from this analysis demonstrate that at the permissive temperature of 30°C, the mutant strains harboring the vector control (pBAD33) and the vector containing the recombinant gene (pBAD33::*murB/C*_*Vs*_) were both able to grow. However, when exposed to the non-permissive temperature of 42°C, only the strains expressing the *murB/C* recombinant gene were able to grow (Figure 4A). This result was corroborated by assessing bacterial growth in liquid medium over a period of 24 h. At 30°C the mutants harboring the vector-only and the *murB/C*− expressing vector grew as expected. The growth phenotype at the non-permissive temperature of 42°C demonstrated that only the mutant strains expressing the *murB/C* gene were able to grow when compared to the vector-only controls (Figure 4B). The lack of growth based on the optical density of the vector-only controls at 42°C can be attributed to rapid lysis of the cells due to the lack of proper peptidoglycan synthesis (Figure 4B). The assessment of crude soluble protein extracts from the complementation experiment using SDS-PAGE analysis confirmed the production of the recombinant MurB/C fusion enzyme (~87.3 kDa) in the mutant backgrounds grown at 42°C that was not present in the extracts from the mutant harboring the vector-only control when both were under inducing conditions using arabinose (Figure 4C). Together, these analyses demonstrated that the recombinant MurB/C fusion enzyme from *V. spinosum* was endowed with the reductase (MurB) and ligase (MurC) activities, and that the amount produced was sufficient to sustain the growth of the *E. coli* mutants.

Attempts to clone and assay the *in vivo* activity of the individual MurB and MurC domains of the MurB/C_*Vs*_ fusion protein were then made. Protein dissection was performed on the basis of the mapping experiments described above, i.e., alignment of the *V. spinosum* protein sequence with that of MurB and MurC ortholog proteins from *E. coli*. These two domains were cloned in the p*Trc*His60 vector, in each case in two versions: with the MurC domain starting at the Met1 residue and terminating either at the Gly477 or the Val481 residue, and the MurB domain starting either at the Gly479 or the Lys482 residue (preceded by a Met residue) and terminating at the last residue (Leu770) of the fusion protein. The two p*Trc*His60::*murC*_*Vs*_ constructs thus generated complemented the thermosensitive *murC* mutant strain H1119, indicating that the shortest version ending at Gly477 clearly exhibited MurC activity. IPTG was not required for complementation, indicating that basal expression of the *murC*_*Vs*_ domain from the p*Trc*His60 vector was sufficient to sustain cell growth and viability of the *murC* mutant at the non-permissive temperature of 42°C (Supplemental Figure 1). However, the two other p*Trc*His60::*murB*_*Vs*_ constructs failed to complement the growth defect of the *murB* mutant strain ST5 and induction of gene expression with 0.5 mM IPTG yielded the same result. The latter finding could be interpreted in several ways: inappropriate design of the *murB* domain initiation codon, physical instability of these truncated forms of the fusion protein, or inability of the MurB domain to function independently (absolute requirement for the presence of the MurC domain). Our data show that this is not the case for the MurC domain whose activity did not depend on the presence of the MurB domain.

### Properties and kinetic parameters of MurC activity from MurB/C*_*Vs*_*

The *in vitro* l-alanine-ligase activity of the MurB/C_*Vs*_ fusion enzyme was revealed using a radioactive assay, which was also used to determine the properties and kinetic parameters (Table 3). The optimal pH and temperature for MurC_*Vs*_ were found to be 9.0 and 44–46°C, respectively. As it is the case for the other Mur ligases (Barreteau et al., 2008), magnesium ions were essential for the activity: the optimal concentration was 10 mM. It was shown that the addition of 10 mM ammonium sulfate and 0.5 mg.mL^−1^ BSA increased the activity by 35 and 55%, respectively. With l-alanine as the amino acid substrate, the apparent *k*_*cat*_ of the enzyme was 480 min^−1^ (*V*_*max*_, ca. 5500 nmol.min^−1^.mg^−1^). Amino acids Gly and l-Ser, which are found at position 1 of the peptide stem in some bacteria (Schleifer and Kandler, 1972; Vollmer et al., 2008), were also tested as substrates (Table 4). l-Ala was the best substrate, which is in agreement with the amino acid composition of *V. spinosum* peptidoglycan (McGroty et al., 2013). However, the difference with the two others was not as important as for the *E. coli* MurC ortholog (Liger et al., 1995).

**Table 3:** **Properties and apparent kinetic parameters of ***V. spinosum*** MurC in comparison with its orthologs from ***E. coli*** and ***C. trachomatis*****.

**Kinetic parameter**	**MurC*_*Vs*_*^a^**	**MurC*_*Ec*_*^b^**	**MurC*_*Ct*_*^c^**
Optimal pH	9.0	8.6	8.0–8.5
Optimal Mg^2+^ concentration (mM)	10	10–20	20
Optimal temperature (°C)	44–46	45	nd^d^
*K*${}_{\text{m}}^{\text{ATP}}$ (μM)	470 ± 160	450	162
*K*${}_{\text{m}}^{\text{UDP}-\text{MurNAc}}$ (μM)	90 ± 25	100	196
*K*${}_{\text{m}}^{\text{L}-\text{Ala}}$ (μM)	25 ± 10	20	124
*V*_max_(nmol.min^−1^.mg^−1^)	5500 ± 50	17300	73.8

a *The apparent kinetic parameters were determined as described in Section Materials and Methods. The concentrations of the fixed substrates were 5 mM for ATP, 1 mM for UDP-MurNAc, and 0.2 mM for l-Ala. The concentration ranges for the varied substrates were 0.2–5 mM for ATP, 25–500 μM for UDP-MurNAc, and 10–400 μM for l-Ala*.

b *From (Liger et al., 1995)*.

c *From (Hesse et al., 2003)*.

d *nd, not determined*.

**Table 4:** **Specificity of MurC_***Vs***_ for the amino acid substrate**.

**Substrate**	**Enzymatic activity (nmol.min^−1^.mg^−1^)^a^**
L-Ala	4900 ± 250
L-Ser	3200 ± 130
Gly	3570 ± 220

a *Determined as described in Section Materials and Methods with fixed concentrations of ATP (5 mM), UDP-MurNAc (1.5 mM), and amino acid (1.5 mM)*.

### Attempts to demonstrate the *In vitro* MurB Activity from MurB/C*_*Vs*_*

Several attempts were made to measure the *in vitro* reductase activity of MurB/C_*Vs*_ with two assays: a spectrophotometric assay using commercial UDP-GlcNAc-EP and NADPH, and a coupled MurA/MurB assay using UDP-[^14^C]GlcNAc and *E. coli* MurA. However, neither decrease of absorbance at 340 nm nor appearance of labeled UDP-MurNAc occurred, even at high protein concentrations. It was checked that the expected reaction took place in both assays when MurB/C_*Vs*_ was replaced by *E. coli* MurB (data not shown). In order to ascertain that the absence of reaction in the coupled assay was not due to strong inhibition by the MurB product, l-alanine, ATP, and Mg^2+^ were added so that the MurC_*Vs*_ activity might displace the reaction toward the right. A new radioactive compound appeared, but its retention time (25 min) was not consistent with that of UDP-MurNAc-l-Ala (20 min) (data not shown). It was presumably the result of the direct addition of l-alanine to UDP-GlcNAc-EP, a reaction that has been shown to occur with *E. coli* MurC (Liger et al., 1995).

### Is MurB/C*_*Vs*_* active as a fusion enzyme *in vivo*?

**Figure 5:**
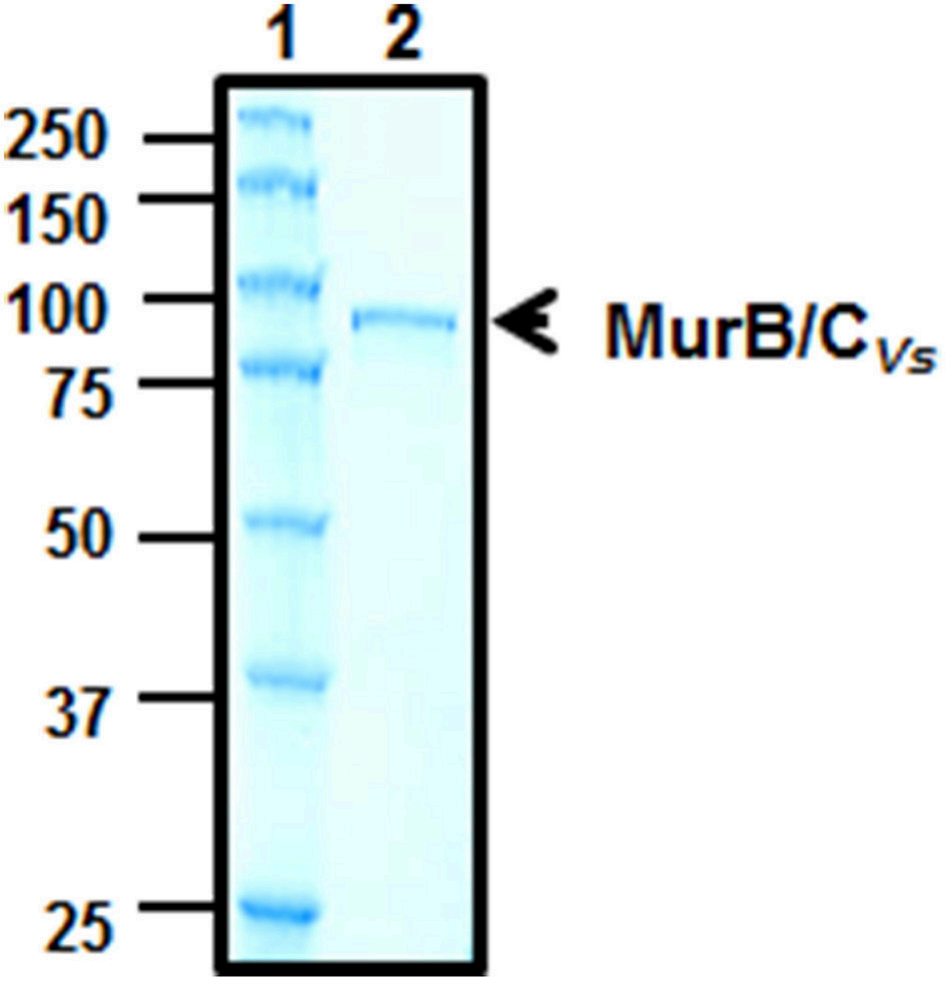
**Purification of MurB/C by affinity chromatography**. Lane (1), protein ladder (kDa). Lane (2), 0.5 μg of purified MurB/C. The proteins were resolved on a 10% (w/v) acrylamide gel and stained with Coomassie blue for visualization.

**Figure 6:**
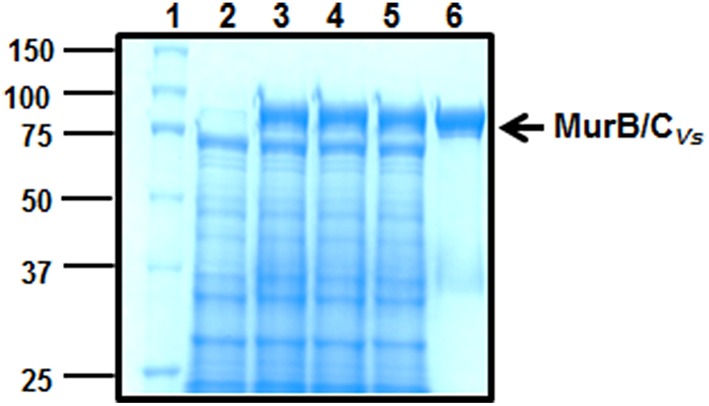
**Assay to assess cleavage of MurB/C using a crude protein extract from ***V. spinosum*****. Lane (1), protein ladder (kDa). Lane (2), 15 μg of *V. spinosum* extract. Lane (3), 15 μg of *V. spinosum* extract plus 7.5 μg of purified MurB/C at time zero. Lane (4), 15 μg of *V. spinosum* extract plus 7.5 μg of purified MurB/C at 60 min. Lane (5), 15 μg of *V. spinosum* extract plus 7.5 μg of purified MurB/C at 120 min. Lane (6), 7.5 μg of purified MurB/C. The temperature of the assay was 30°C. The proteins were resolved on a 10% (w/v) acrylamide gel and stained with Coomassie blue for visualization.

The SDS-PAGE showing the expression of the MurB/C_*Vs*_ in the *murB* and *murC* mutant backgrounds demonstrated that the recombinant enzyme was not cleaved in *E. coli* (Figure 4C). However, given the fact that there is a 100-residue linker region between the MurC and MurB domains (Figure 3), we were interested in answering the question of whether the fusion enzyme is cleaved in *V. spinosum*. This would indicate that after translation, the enzyme is processed by a protease to create two separate and distinct polypeptides of MurB and MurC. To answer this question, the purified recombinant enzyme (Figure 5) was used in a cleavage assay using crude soluble protein extract from *V. spinosum*. The assay showed that the recombinant enzyme was not cleaved when incubated with an extract from *V. spinosum* over a period of 120 min (Figure 6). It should be noted that the result was consistent when the assay was done using several concentrations of *V. spinosum* protein extract of up to 15 μg and several buffer systems with varying pH values (data not shown). Based on the complementation, and supported by the cleavage assay, it is probable that the enzyme is active as a fusion enzyme in *V. spinosum*. Fusion enzymes involved in PG biosynthesis are not a new phenomenon; a MurC/Ddl fusion enzyme from *Chlamydia trachomatis* has been characterized and it was shown that the d-Ala:d-Ala ligase activity of the Ddl domain is dependent on the fusion structure of the MurC/Ddl protein (McCoy and Maurelli, 2005). Our present data show that at least the MurC domain of the *V. spinosum* MurB/C fusion protein is functionally independent as its expression allowed complementation of a temperature-conditional *murC* defect in *E. coli*.

### Unusual MurB and MurC composition is prevalent in the currently sequenced members of verrucomicrobia

**Figure 7:**
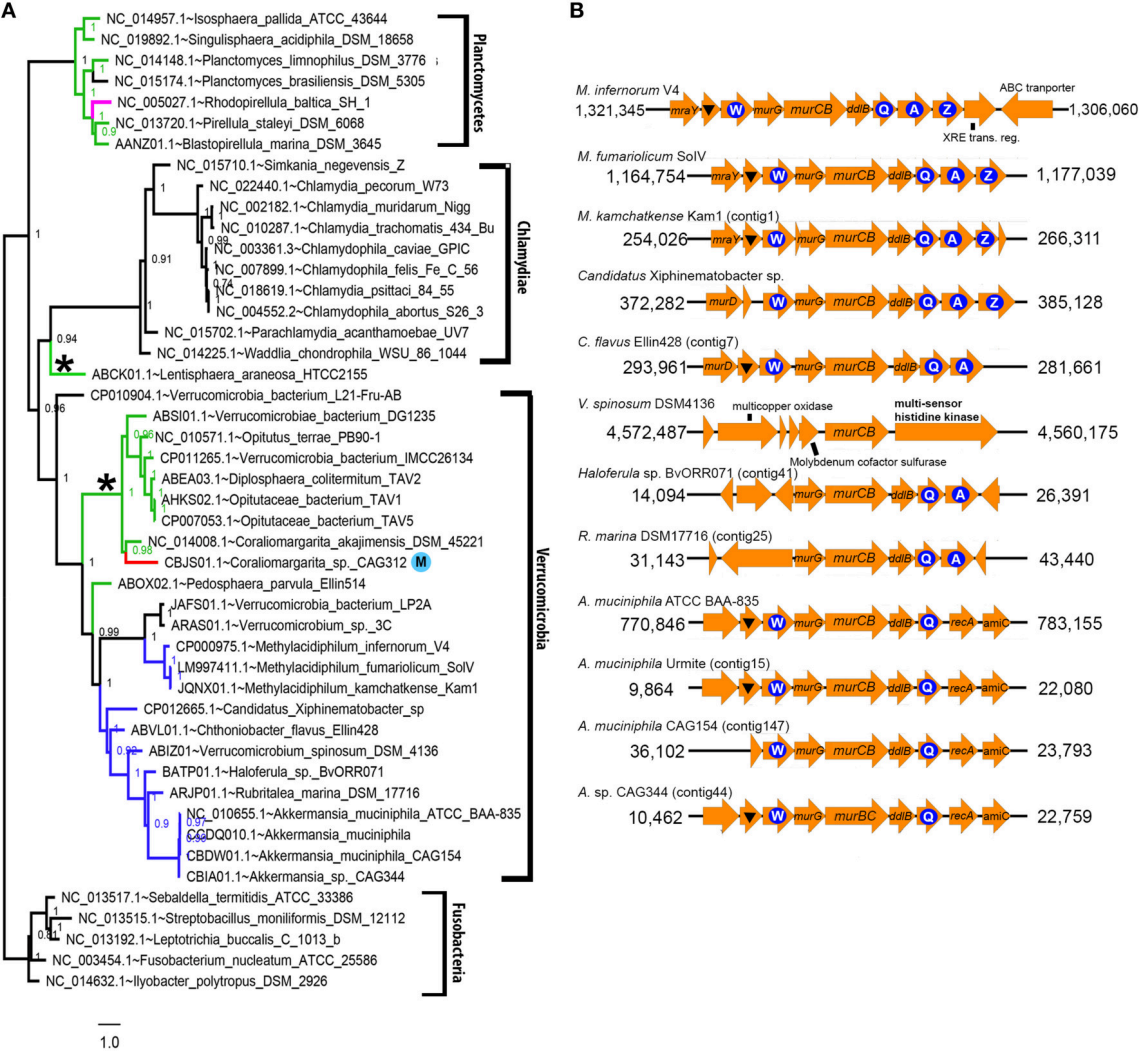
**(A)** Species-tree of the Planctomycetes, Verrucomicrobia, and Chlamydiae (PVC) clade constructed using 400 universal proteins. Values next to each branch node represent SH-like branch supports as calculated by FastTree2 (Price et al., 2010). The scale bar indicates the number of substitutions per site. The tree was rooted with members from the phylum Fusobacteria as outgroup. Asterisk sign indicates lineage possessing a longer than usual MurB protein (>600 amino acid residues) and blue circle with the letter “M” indicates highly-fragmented genome that was derived from metagenomic assembly. The color designations for colored taxons are as follows: red for missing MurB and MurC, pink for missing MurC and MurB detected at slightly below the TIGR00179 trusted-cutoff score of 144.5 (142 > χ > 144.5), green for missing MurC and blue for fused MurB/C. **(B)** Gene neighborhood of fused *murB/C* ORFs. Blue circle represents *fts* gene and letter within the circle represents the respective *fts* gene component. LysM-containing protein coding genes are indicated with a triangle and genes without label code for hypothetical proteins. Contig numbers are indicated next to species with draft genome sequence.

PhyloPhlAn-generated species tree of the PVC clade supports the monophyletic placement of the major lineage (SH-likelihood branch support of >0.90). A slightly lower than average SH-likelihood support value of 0.94 was observed in the branch splitting the Chlamydiae and Lentisphaerae clades which presumably is due to low taxon sampling (*N* = 1) of the Lentisphaerae clade (Figure 7A). Fused MurB/C is present in all the currently sequenced members of Verrucomicrobiales (9/9) and Methylacidiphilales (3/3). These members usually exhibit a conserved *ftsW-murG-murB/C*-*ddlB-ftsQ* gene organization (Figure 7B) with the exception of *V. spinosum* DSM 4138, the type species and type strain for the genus *Verrucomicrobium* and species *V. spinosum*, respectively. In *V. spinosum* DSM 4138, the *murB/C* gene is flanked by genes that are not directly related to cell-wall synthesis. However, the monophyletic grouping of MurB/C protein (including that of *V. spinosum* DSM 4138) suggests that fused MurB/C is an authentic molecular signature in specific lineages of the phylum Verrucomicrobia and is not a result of inter-phylum horizontal gene transfer (Supplemental Figures 2A,B). TIGRFAM search failed to detect MurC domain in the predicted proteome from members of the class Opitutae (Figure 7A, green-colored branch). Despite lowering the detection threshold limit for MurC to the least stringent limit, e.g. noise cutoff, the MurC domain still could not be detected, thus supporting the absence of authentic MurC domain in this lineage. However, it is worth noting that the MurB proteins detected in nearly all members from Opitutae are longer than usual and contain the Mur ligase domain located at the N-terminal (Supplemental Figure 3 and data not shown), suggesting that although the N-terminal region has diverged substantially from the typical MurC, it may still share partially overlapping function with MurC.

## Discussion

In the present paper, the *in vivo* MurB and MurC activities of the MurB/C_*Vs*_ fusion enzyme were revealed by functional complementation experiments. Furthermore, the protein was shown to be endowed with MurC ligase activity *in vitro* through a specific radioactive assay. In Table 3, the properties and kinetic parameters of the MurC_*Vs*_ activity are compared with those of a reference ortholog (*E. coli*) and a phylogenetically related ortholog (*C. trachomatis*). The enzymes from *V. spinosum* and *E. coli* are comparable regarding their kinetic parameters. The maximum velocity of MurC_*Vs*_ is three-fold lower (but the *k*_*cat*_ value is only two-fold lower due to the high molecular mass of the fusion enzyme) when compared to the *E. coli* MurC. In addition, the optimal pH value (9.0) of the MurC_*Vs*_ falls within the range found for most Mur ligases (8.0–9.2) (Patin et al., 2009) and the observed pH optimum is comparable to that of MurE from *V. spinosum* (9.6). It is possible that such values of pH ≥9 reflect environmental factors such as the natural habitat of *V. spinosum* (McGroty et al., 2013). On the other hand, MurC enzymes from *V. spinosum* and *C. trachomatis* have quite different kinetic parameters. The main difference is the maximum velocity, which is 75-fold lower for *C. trachomatis* MurC when compared to the MurC from *V. spinosum*. Although many reasons can be put forward to explain such a low maximum velocity, a physiological explanation might be related to the slower growth rate of *C. trachomatis* given the fact that it is an intracellular parasite (Hesse et al., 2003).

Although l-alanine is the best substrate *in vitro*, l-serine and glycine are also reasonable substrates (Table 4). From the *in vitro* data, the discrimination between the three amino acids *in vivo* is much less obvious than for MurC from *E. coli* (Liger et al., 1995). Nevertheless, the determination of the amino acid composition of PG from *V. spinosum* proves undoubtedly that l-Ala is the amino acid found at position 1 of the peptide stem (McGroty et al., 2013). With *C. trachomatis* MurC, the discrimination was even less obvious, since the three amino acids were added to UDP-MurNAc with similar *V*_*max*_*/K*_*m*_ values, thereby preventing us from deducing which amino acid was present in the putative chlamydial PG (Hesse et al., 2003; Patin et al., 2012). The recent mass spectrometric detection, from *C. trachomatis*-infected cell lysates, of muropeptides with alanine or glycine at position 1 strongly suggests that these amino acid are both used as MurC substrates by *C. trachomatis in vivo* (Packiam et al., 2015).

While the MurC_*Vs*_ activity could be totally characterized, the MurB_*Vs*_ activity could not be detected *in vitro*, even with the use of two different assays. Attempts to modify the assay conditions (different pH value, NADH instead of NADPH) were unsuccessful. A plausible explanation for the lack of MurB_*Vs*_ activity could be the denaturation of the MurB domain during or after the purification steps. Even though a cryoprotectant was included in the storage buffer, such a phenomenon cannot be ruled out. Another explanation would be that an unidentified cofactor(s) is necessary for activity which we are not aware of at this time. Nevertheless, the functional complementation of the *E. coli murB* thermosensitive strain by the *murB/C*_*Vs*_ gene proves that the MurB_*Vs*_ domain is active in *in vivo* conditions.

Based on phylogenetic and protein domain scanning, most members of the Verrucomicrobia possess different compositions of MurB and MurC proteins. In some lineages, both proteins are fused as is the case for *V. spinosum*. In others, the MurC seems to be absent. Given the phenotype of Verrucomicrobia regarding the unusual body plan with respect to wart-like and projections radiating from the central body, one would expect that PG biosynthesis would have an integral role pertaining to the shape of the bacterium (McGroty et al., 2013). Theoretically, the possession of a fusion protein that catalyzes sequential steps in a particular pathway may constitute an advantage when compared to a system where these orthologous proteins are separate. These advantages may include control of the expression of two genes as a single unit, substrate channeling due to proximity of the catalysts, higher catalytic activities, etc. Whether such an advantage, if it exists, influences growth and development of *V. spinosum* is currently not known. Further studies to determine the structure of the MurB/C fusion enzyme would help shed some light regarding the catalytic properties of the enzyme, especially since we were not able to demonstrate the MurB activity.

In summary, we present the first description of a MurB/C fusion enzyme from *V. spinosum*. *In vitro* biochemical analyses demonstrated that the enzyme is capable of catalyzing the ligase (MurC) reaction at position 1 on the peptide stem. *In vitro* analyses to demonstrate the MurB reductase were not successful even though *in vivo* analyses demonstrated that the fusion gene is able to functionally complement *E. coli* strains that harbor mutations in the *murB* and *murC* genes. Furthermore, dissection experiments showed that the MurB domain of the fusion protein was not essential for the UDP-*N*-acetylmuramate::l-alanine ligase activity of the MurC domain. As this is the first description of a MurB/C fusion enzyme, there are no structural studies in the literature. Therefore, studies to solve the structure of fusion enzymes such as MurB/C_*Vs*_ and MurC/Ddl from *C. trachomatis* would help elucidate properties of the enzyme and have the potential to provide information regarding the rational design and/or identification of compounds that are deemed inhibitory and that could be developed as antibiotics. As such, this study lays the foundation regarding the further understanding of the kinetic, physical, and structural properties of a novel enzyme involved in the synthesis of PG.

## Author contributions

All authors listed, have made substantial, direct and intellectual contribution to the work, and approved it for publication.

### Conflict of interest statement

The authors declare that the research was conducted in the absence of any commercial or financial relationships that could be construed as a potential conflict of interest.

## Supplementary material

Supplementary Figure 1 **Growth of *E. coli murC* thermosensitive strain H1119 transformed by vector p*Trc*His60 (A), p*Trc*His60::*murC*_*Ec*_ (B), p*Trc*His60::*murC*_*Vs*_-1 (C), and p*Trc*His60::*murC*_*Vs*_-2 (D)**.

Supplementary Figure 2 **Maximum likelihood tree of (A) MurB and (B) MurC proteins**. Both trees are constructed using IQ-TREE version 1.3.10 with the optimized LG+G4 model. Value next to branch corresponds to IQ-TREE ultrafast bootstrap support value. Taxons containing fused MurB/MurC are colored red. The scale bar indicates the number of substitutions per site.

Supplementary Figure 3 **Complete InterProScan analysis of longer-than-usual MurB protein identified from the draft genome of Verrucomicrobiae bacterium DG1235 (accession number: ABSI01)**. The MurC domain corresponding to InterProScan ID IPR005758 (HAMAP: MF_00046; TIGRFAMs: TIGR01082) was absent from this MurB protein. However, domains corresponding to a general Mur ligase (N-terminal catalytic, central, and C-terminal domains) are present in the N-terminal region.
