# Supplementary figures and images for: Identification and Partial Characterization of a Novel UDP-N-Acetylenolpyruvoylglucosamine Reductase/UDP-N-Acetylmuramate:l-Alanine Ligase Fusion Enzyme from Verrucomicrobium spinosum DSM 4136T
Source: Front Microbiol. 2016 Mar 23;7:362. doi: 10.3389/fmicb.2016.00362 (PMC4803751; doi:10.3389/fmicb.2016.00362)

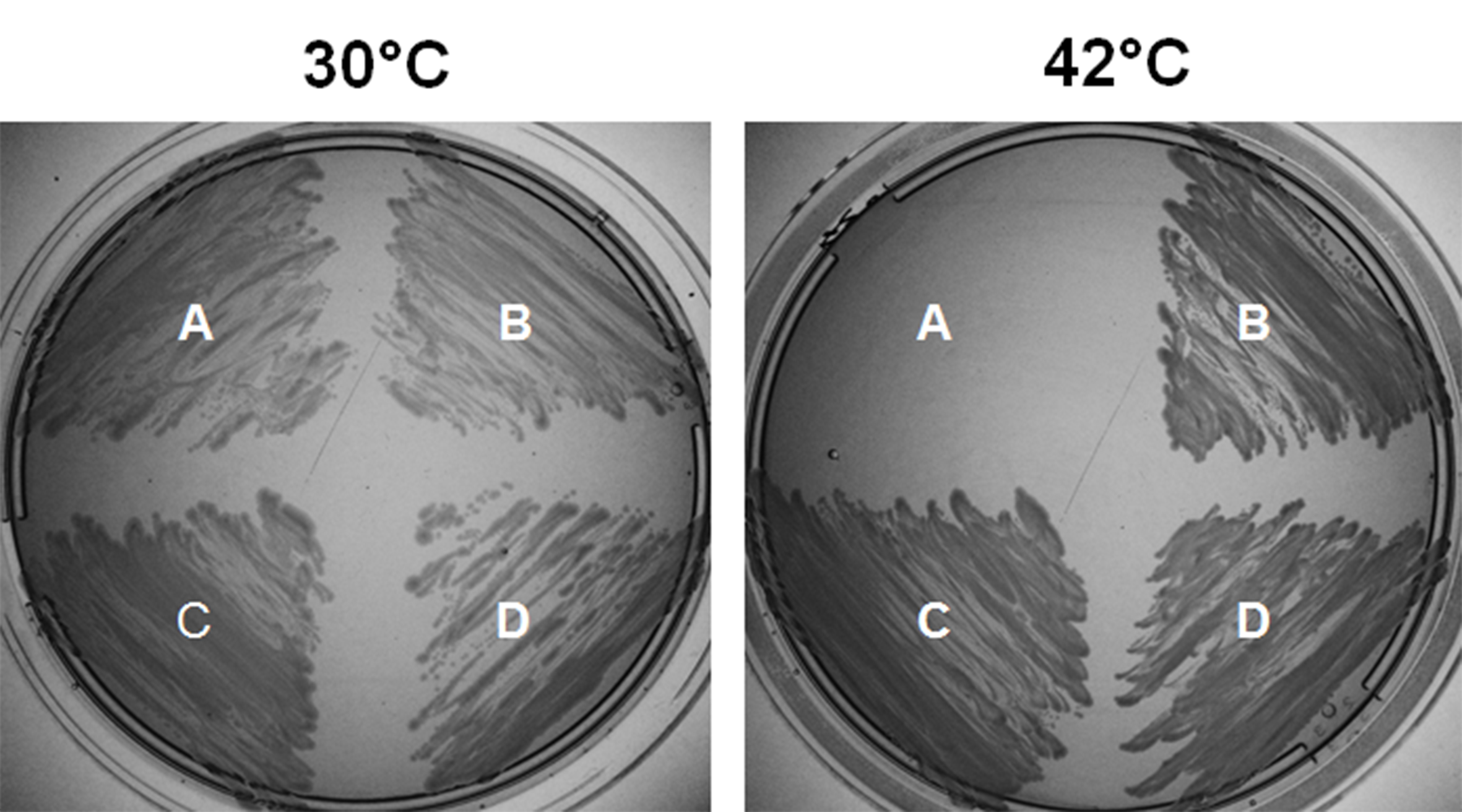

Supplement: Supplementary Figure 1 — Growth of E. coli murC thermosensitive strain H1119 transformed by vector pTrcHis60 (A), pTrcHis60::murCEc (B), pTrcHis60::murCVs-1 (C), and pTrcHis60::murCVs-2 (D). [file Image1.TIF]

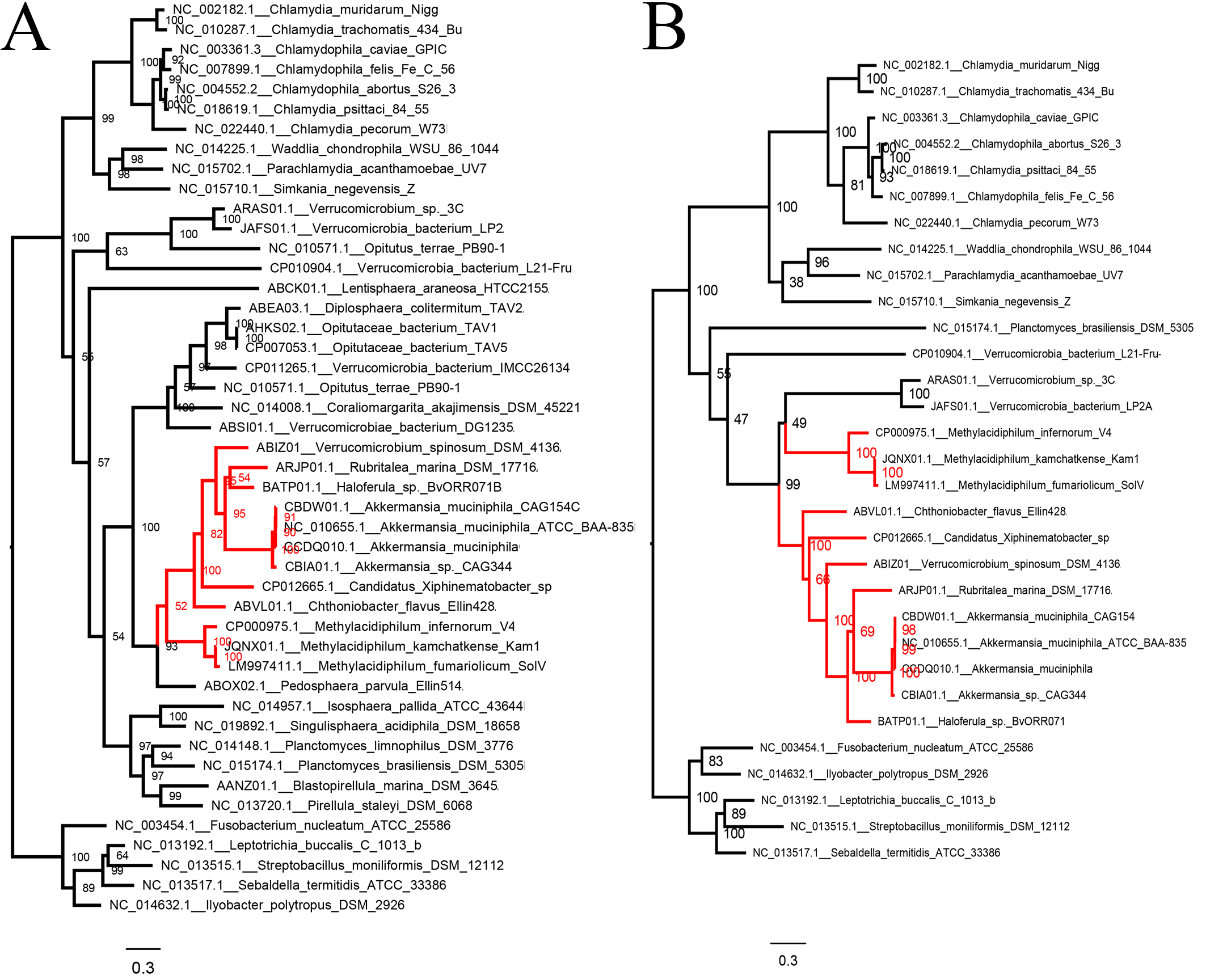

Supplement: Supplementary Figure 2 — Maximum likelihood tree of (A) MurB and (B) MurC proteins. Both trees are constructed using IQ-TREE version 1.3.10 with the optimized LG+G4 model. Value next to branch corresponds to IQ-TREE ultrafast bootstrap support value. Taxons containing fused MurB/MurC are colored red. The scale bar indicates the number of substitutions per site. [file Image2.TIF]

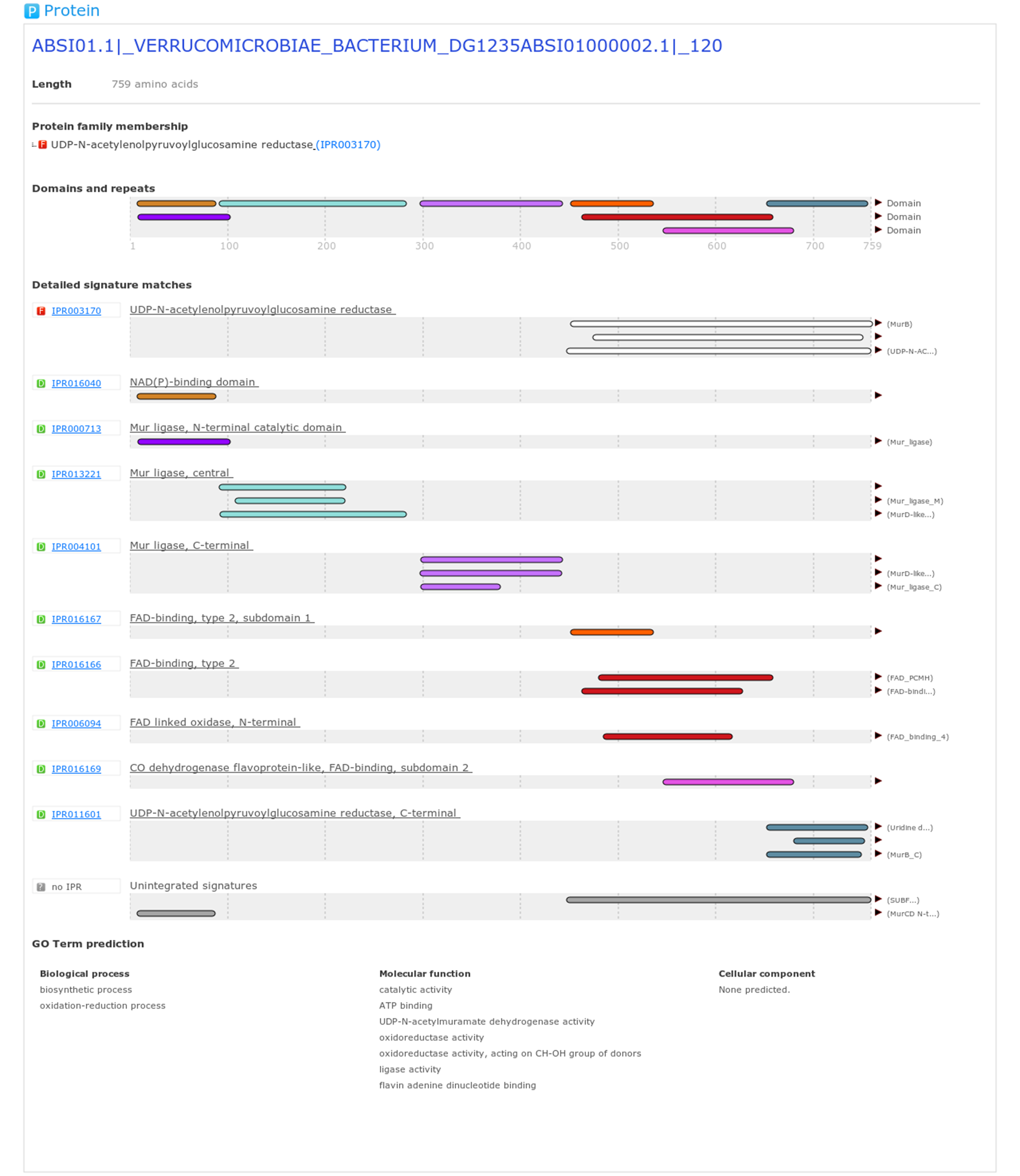

Supplement: Supplementary Figure 3 — Complete InterProScan analysis of longer-than-usual MurB protein identified from the draft genome of Verrucomicrobiae bacterium DG1235 (accession number: ABSI01). The MurC domain corresponding to InterProScan ID IPR005758 (HAMAP: MF_00046; TIGRFAMs: TIGR01082) was absent from this MurB protein. However, domains corresponding to a general Mur ligase (N-terminal catalytic, central, and C-terminal domains) are present in the N-terminal region. [file Image3.TIF]
